# Twenty-four hour quantitative-EEG and *in-vivo* glutamate biosensor detects activity and circadian rhythm dependent biomarkers of pathogenesis in Mecp2 null mice

**DOI:** 10.3389/fnsys.2014.00118

**Published:** 2014-06-27

**Authors:** Michael V. Johnston, Simon Ammanuel, Cliona O'Driscoll, Amy Wozniak, Sakkubai Naidu, Shilpa D. Kadam

**Affiliations:** ^1^Neuroscience Laboratory, Departments of Neurology and Pediatrics, Hugo Moser Research Institute at Kennedy Krieger, Johns Hopkins University School of MedicineBaltimore, MD, USA; ^2^Neuroscience Laboratory, Hugo Moser Research Institute at Kennedy KriegerBaltimore, MD, USA; ^3^Department of Environmental Health Sciences, Johns Hopkins Bloomberg School of Public Health, Hugo Moser Research Institute at Kennedy Krieger, Johns Hopkins University School of MedicineBaltimore, MD, USA; ^4^Biostatistics Center, Johns Hopkins Bloomberg School of Public Health, Johns Hopkins University School of MedicineBaltimore, MD, USA; ^5^Departments of Neurology and Pediatrics, Hugo Moser Research Institute at Kennedy KriegerBaltimore, MD, USA; ^6^Neuroscience Laboratory, Departments of Neurology, Hugo Moser Research Institute at Kennedy Krieger and Johns Hopkins University School of MedicineBaltimore, MD, USA

**Keywords:** Mecp2, sleep structure, glutamate, biomarkers, Rett syndrome

## Abstract

Mutations in the X-linked gene encoding methyl-CpG-binding protein 2 (Mecp2) cause most cases of Rett syndrome (RTT). Currently there is no cure for RTT. Abnormal EEGs are found in 100% of RTT cases and are associated with severe sleep dysfunction, the cause of which is not well understood. Mice deficient in MeCP2 protein have been studied and characterized for their neuropathological and behavioral deficits to better understand RTT. With the goal to study the non-ictal EEG correlates in symptomatic Mecp2 KO mice (Mecp2^tm1.1Bird/y^), and determine novel EEG biomarkers of their reported progressive neurodegeneration, we used 24 h video-EEG/EMG with synchronous *in-vivo* cortical glutamate biosensor in the frontal cortex. We scored the EEG for activity states and spectral analysis was performed to evaluate correlations to the synchronous extracellular glutamate fluctuations underlying Mecp2 inactivation as compared to WT. Significant alterations in sleep structure due to dark cycle-specific long wake states and poor quality of slow-wave sleep were associated with a significant increase in glutamate loads per activity cycle. The dynamics of the activity-state-dependent physiological rise and fall of glutamate indicative of glutamate homeostasis were significantly altered in the KO mice. Colorimetric quantitation of absolute glutamate levels in frontal cortex also indicated the presence of significantly higher levels in KO. This study for the first time found evidence of uncompensated sleep deprivation-like EEG biomarkers that were associated with glutamate homeostatic dysfunction in the Mecp2 KO mice.

## Introduction

Rett syndrome (RTT) is an X-linked, neurodevelopmental disorder which is a common cause for genetic mental retardation in girls, one in every 8500 live births. The gene defect in males usually results in severe clinical manifestations and fatality. Infants with Rett syndrome seem to grow and develop normally for 6–18 months, but then regress developing and lose skills and abilities. Autistic-like behaviors also commonly appear at this stage. Mutations in the gene encoding Mecp2 (methyl-CpG-binding protein 2) are known to cause the majority of cases (90–95%, Neul et al., 2008) of typical RTT and are also found in neuropsychiatric syndromes like autism, bipolar disorder with cognitive deficits and childhood-onset schizophrenia with intellectual disabilities. Currently there is no cure for RTT. Epilepsy is a major co-morbidity [60–80%, however abnormal EEGs are found in 100% of RTT cases (Glaze et al., 1998; Hagberg, 2002; Moser et al., 2007; Glaze et al., 2010; Pintaudi et al., 2010)]. Sleep dysfunction is commonly reported (Young et al., 2007). However, little is known about the mechanisms by which chronic sleep dysfunction modulates the severity of temporal deterioration in the regression stage in RTT. Video-EEG monitoring has been suggested to provide definitive information regarding the need for therapy in RTT (Glaze et al., 1998).

Mecp2 has many functions and its roles as a transcriptional repressor, activator, and RNA-binding protein have been examined. Although the responsible gene was identified more than a decade ago (Amir et al., 1999), the mechanisms underlying the RTT symptomatology are poorly understood. Mice with *Mecp2* mutations show neuropathological and behavioral deficits similar to that reported for RTT (Chen et al., 2001; Guy et al., 2001; Shahbazian et al., 2002; Chao et al., 2010; De Filippis et al., 2010). Studies using different strains of *Mecp2* mutants have identified several impairments that likely contribute to the pathophysiology of the disorder (Shahbazian et al., 2002; Lawson-Yuen et al., 2007; Jentarra et al., 2010); however the role of Mecp2 inactivation in sleep and glutamate homeostasis has not been investigated. Additionally minimal information exists regarding how the loss of MeCP2 protein affects network activity in the brain (Glaze, 2004). Quantitative EEG (qEEG) with synchronized *in-vivo* glutamate recordings can provide a valuable tool to evaluate the CNS network modulation of recently investigated therapeutic interventions that have shown reversal of respiratory and behavioral phenotypes (Nag and Berger-Sweeney, 2007; Abdala et al., 2010; De Filippis et al., 2012).

Due to the high penetrance of MECP2 mutations in humans, at least some phenotypes that mimic symptoms of human RTT are common in the male KO Mecp2^tm1.1Bird/y^ mice unlike the female HET mice that do not become symptomatic until they are adults. This is reflected in the overall bias of the scientific community, which has performed most phenotypic analyses in the male hemizygous mice. In spite of rigorous neuroanatomical, behavioral, motor and cognitive testing in the mutant males in this model, no studies have investigated or quantitated their sleep dysfunction (Katz et al., 2012) which is a prominent feature in RTT patients. In a recent NIH workshop organized to recognize gaps in RTT preclinical studies, one of the highlighted goals was to characterize the phenotypes that represent significant challenges for medical management of individuals with RTT, including seizures, and gastrointestinal, cardiorespiratory, sensory and sleep-wake problems (Katz et al., 2012). Sleep dysfunction often occurs in RTT and is considered a major problem (Young et al., 2007). High rates of comorbidities of epilepsy and sleep dysfunction with genetic disorders like RTT, autism, tuberous sclerosis, and fragile X suggest potentially shared underlying mechanisms (Malow, 2004). The precise mechanism by which loss of MeCP2 function in RTT results in these comorbidities remains uncertain. Therefore, to investigate the role of Mecp2 X-inactivation on sleep and glutamate homeostasis, this study, for the first time, used *in-vivo* synchronous video-EEG/EMG and glutamate biosensing (Figure S1A) in symptomatic Mecp2 KO mice. The goal was to identify definitive quantitative-biomarkers, both in the 24 h EEG and brain glutamate levels of the KO mice, and investigate their association with symptomatology during rapid progression of pathophysiology. This study reports the novel findings of these experiments.

## Methods

The present study utilized a mouse model of Mecp2 deficiency developed by Jacky Guy and Adrian Bird (Guy et al., 2001). Our colony consists of Mecp2^tm1.1Bird^ mice (Jackson Laboratory, Bar Harbor, Maine) on a C57BL/6 background (heterozygote backcrossed with C57BL/6 males for at least nine generations). The Bird Mecp2-deficient mice were bred and housed behind a barrier in the animal facilities at Johns Hopkins Medical Institutions with a standard 12-h light on/off cycle with food and water available *ad-libitum*. These facilities meet Federal and NIH regulations for animal care and are accredited by the American Association for Accreditation of Laboratory Animal Care. All animal experimentation was conducted in accordance with protocols approved by the guidelines of the Johns Hopkins of Animal Care.

For genotyping, tail-clipping samples were obtained between P4 and P7. The samples were processed using a DNAeasy (Young et al., 2007) Blood & Tissue Kit from Qiagen for genotyping, as previously published (Metcalf et al., 2006; Blue et al., 2011)

The *Mecp2*^tm1.1Bird^ (KO; *n* = 6) and wild-type mice (WT; *n* = 6, 5 males, 1 female, littermates; no male WT littermate was available for one pair; six paired recordings) used in this study were all aged between 6–7 weeks. Under general anesthesia, mice were surgically implanted with electroencephalograph [EEG; sub-dural electrodes modified from a similar previous protocol used in rats for chronic EEG recording (Kadam et al., 2010)] and electromyograph (EMG; supra-scapular) recording electrodes along with a biosensor guide cannula stereotaxically implanted in the M1 region of the frontal cortex (A/P + 2.1, M/L+1.0, DV −1.0) (Figures S1A,B). After recovery from surgery (3–7 days), a glutamate biosensor (Figure S1A) was inserted and glutamate levels were sampled at 1 s intervals. EEG and EMG waveforms along with continuous glutamate levels were recorded for an entire 24 h light-dark cycle.

### Electrode calibration

After recording for 24 h, the glutamate biosensor was explanted under anesthesia and a post-recording calibration performed according to the manufacturers' guidelines (Pinnacle Technologies Inc., Lawrence, KS, USA). Post-calibration glutamate readings (Figure S1C) were used to convert the *in-vivo* electrical readout for relative change in glutamate concentrations in μAMPs into glutamate concentrations in μM (Figure S1C—inset). Sensitivity of the glutamate biosensor *in-situ* was tested by injecting MK801 intra-peritoneal injection (5 mg/kg), which elicited an immediate (<1 min) and significant rise in the glutamate reading, by the sensor (Figure S1D). MK801 which is an NMDA receptor antagonist is known to induce increases in endogenous glutamate levels (Wyckhuys et al., 2013) and has been used to model various neurologic disorders, such as epilepsy, schizophrenia, and Parkinson disease where abnormal glutamate transmission is hypothesized to be involved (Roenker et al., 2012).

### Data analysis

EEG and EMG waveforms were recorded and analyzed using the 8400 system recording system and Sirenia sleep software package (Pinnacle Technology Inc., Lawrence, KS). All recorded waveforms were scored as 10 s epochs of WAKE, no-rapid eye movement (NREM) or rapid eye movement (REM) sleep by a blinded scorer using synchronous EMG and video for confirmation of wake-inactive states. In 10 s epochs where transitions between behavioral states occurred, the EEG epoch was scored as the EEG state that was >5 s (dominant) within that epoch which also correlated beautifully with EEG and EEG power for each epoch (see Figure S2). Glutamate levels were averaged over each 10 s epoch and correlated with the sleep/wake state after blinded scoring was complete. Changes in glutamate concentration were calculated following post-calibration of individual sensors (Figure S1C).

### *In-vivo* glutamate recorded trace quantitation

Twenty-four hour glutamate recordings showed an initial *in-vivo* exponential drop calibration phase of 2–4 h as the recordings reached a baseline level in each animal. Additionally, during the 24 h continuous recordings some traces showed linear drift contamination of the physiological data. To acquire accurate quantitation of glutamate loads for these periods, the data underwent recalibration for artifact removal. Smoothing splines were applied to the calibration period and the follow-on linear drift contamination of the glutamate traces to prevent overestimation of glutamate loads due to drifting baselines. The calibration period, that began as the biosensor was first inserted *in vivo* and lasted 2–3 h, showed sharp drops in the 1st hour. Therefore, the first hour of readings were not quantitated. Cubic smoothing splines were applied to readings after 1 h through the end of the calibration period. The ends of calibration periods for *in-vivo* stabilization of readings were selected blinded to EEG/EMG data based on stable glutamate recordings without an exponential drop. In addition, linear drift was apparent during certain time periods for individual traces in the post-calibration period. Time periods for apparent linear drift in *in-vivo* glutamate trace recordings were marked for correction blinded to behavioral states (EEG/EMG). Again, cubic smoothing splines were applied to drift periods by an investigator blinded to EEG activity scores. The degree of smoothing was dependent on amount of calibration and drift present in the glutamate trace irrespective of the associated synchronous EEG/EMG recording which was reviewed and scored (blinded to genotype). The mean of the smoothed calibration and drift pieces were then matched to the mean of the original readings.

### Area under the curve method to quantitate glutamate loads per wake cycle

The 24 h traces for glutamate recordings were scatter plots of values recorded per second in real time. Since the *in-vivo* relative glutamate concentrations showed ultradian cycling tied to activity states where peaks matched wake states on EEG and troughs marked sleep, glutamate loads per wake cycle were calculated as area between 2 troughs. Automated computations calculated the area under the curve for each wake cycle. The 2 time points of the cycle were represented by the 2 lowest points of the sleep cycle previous to and following a wake cycle. Area under curve was then calculated using the 1 s trapezoidal rule between those points. All values reported here were calculated by an automated method using a code written in R stats (http://www.r-project.org/).

The methods to quantitate REM glutamate loads (area under the curve) were similar to the overall wake glutamate load cycle calculations. Using the manually scored EEG for identifying episodes of REM in both KO and WT, an automated code detected the lowest points in the EEG synchronous glutamate recording before and after each REM cycle. Since the REM cycles were short duration episodes (10's of seconds) associated with short and sharp rises in glutamate levels during the trough period, we calculated the area under each REM-cycle-associated glutamate peak using time points 10 s before and after the start and end of each REM cycle that was at least ≥30 s long. All values reported here were calculated by an automated method using a code written in R stats (http://www.r-project.org/).

### Percentage rate change of glutamate with change in activity state (sleep-wake)

In order to calculate the percentage rate of change of glutamate following wake or sleep, the 1 min values after the first 10 s EEG epoch scored as wake/sleep was averaged to avoid skewed values due to artifacts. The same was done for another 1 min timeslot recorded 5 min later. Once those numbers were calculated, a simple percentage change, which was the difference in the two calculated average numbers, divided by the first average was multiplied by 100 [% rate change GLU = (2nd value–1st value/1st value)^*^100]. The values analyzed here were also generated by an automated code written in R stats (http://www.r-project.org/).

Percentage rate change of glutamate per REM cycle was calculated similar to Wake and Sleep. Since the REM cycles last only for a few epochs of 10 s each, to calculate percent rate of change of glutamate associated with each REM cycle, we used the average glutamate values of first 3 s of the first REM epoch in each REM cycle, then went 5 s further and calculated the average of the 8–10th seconds of that epoch. The percentage change formula was then applied to these two values to find the rate change.

### *In-vitro* absolute brain glutamate measurement

Fresh frozen harvested brain tissue from EEG recorded and naïve WT and KO mice were resuspended in PBS and sonicated briefly, the suspension was then spun at 10,000 rpm for 30 s and the supernatant collected. Glutamate was measured in a 96-well microplate with a final volume of 100 μl reaction mixture. Reaction mixture contained 20 μl of sample and 70 μl comprised of 50 μM Amplex Red (Invitrogen), 0.04 U/ml glutamate oxidase (Sigma), and 0.125 U/ml HRP (Sigma), in 100 mM Tris–HCl, pH 7.5. Following incubation at 37°C for 10 min, the absorbance of the reaction mixtures were measured at 560 nm. Background absorbances were subtracted from each sample, using the sample incubated with an inactivated enzyme mixture. A standard curve was constructed ranging from 100 to 3 uM of L-glutamic acid. Protein concentrations for the brain samples were calculated by use of the Bradford method with a BSA standard curve.

### Behavioral activity scoring of synchronous video recording

After the 24 h EEG had been scored and wake periods quantified, the synchronous video was quantitated for behavioral activity for 1 h wake slots both during the light and dark periods. For the light cycle wake activity on the next morning of 24 h recording were scored to avoid phase of acclimation to the recording environment. If there were more than one 1 h long wake-cycle, then 1 h from the longest wake cycle was analyzed using the highest score detected during 5 min blocks of video recorded behavioral activity. Scores were graded as follows: 1. Inactive-wake but resting; 2. lightly active-grooming behaviors; 3. medium active—eating+grooming; 4.active- walking, exploring; and 5. Hyperactive—running+wet-dog shakes+rearing.

### Statistics

All quantitative data analyses of glutamate traces was generated using automated algorithms written in R stats. Effects of light and dark phases (diurnal) for KO and WT on duration of sleep state cycles and % rate change were estimated with linear mixed models with a random mouse effect and interaction of cycle and type. Independent sample t-tests (Mann-Whitney U) were used for direct comparisons of quantitative data between the two genotypes for 24 h glutamate loads and sleep structure data. Sleep cycle quantitative data was exported from the Sirenia sleep analysis module after blind scoring of EEG. *F*-tests were used to compare the equality of two variances between group data. For comparing the correlative strength between two groups, Pearson's product moment correlation coefficient, a determiner of linear dependency between two variables, was employed. Significance was set at *p* < 0.05. Mean and standard error of the mean are presented throughout the text and figures.

## Results

### Sleep cycle dysfunction

**Figure 1:**
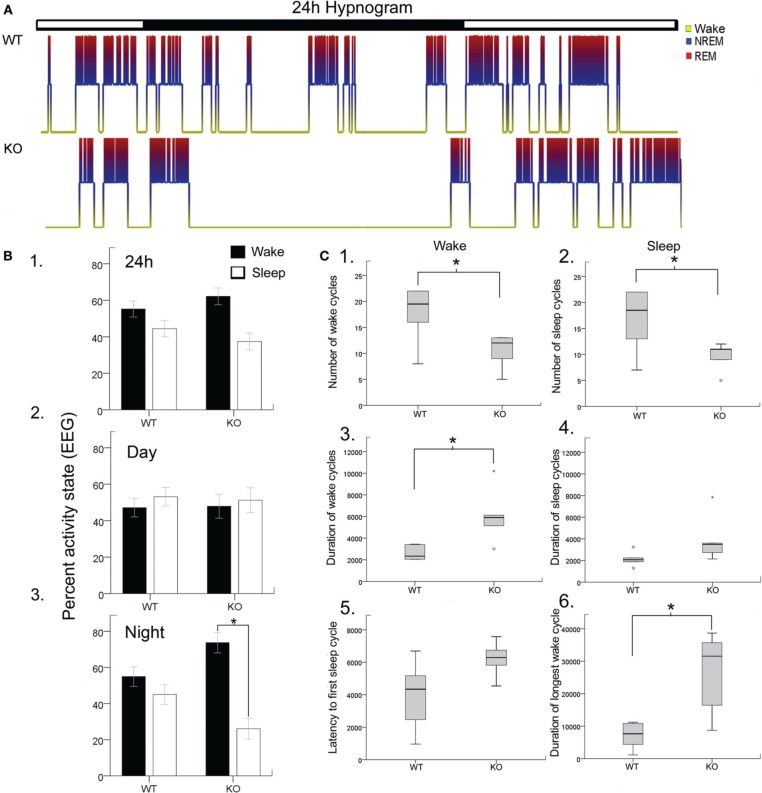
**Blinded video-EEG/EMG manual scoring quantitated activity cycles to generate (A)**. Hypnograms for each mouse in the study and showed significant sleep structure anomalies in the KO mice **(B)**. Histograms quantitating sleep/wake cycles for the data set (WT and KO *n* = 6 each) showed **(B1)**. No significant differences in percent time spent awake or sleeping were found when light/dark cycles (i.e., 24 h) were pooled together (lights on 7 am off 7 pm). However, when the data was split by light/dark cycle **(B2,B3)**. KO mice showed significant sleep deprivation compared to WT mice. The dark cycle longer wake periods were not associated with any significant increase in behavioral quantitation for hyperactivity scores. **(C)** Wake and sleep cycle quantitation revealed an altered number and duration of cycles with significantly long wake cycles in KO mice. **(C1,C2)** Show significantly lower counts of both wake and sleep cycles in KO mice. **(C3,C4)** showed that the wake cycles in KO mice were significantly longer in duration and while the sleep cycles showed a trend to be longer than in WT mice. **(C5)** The latency to first sleep cycle was significantly longer in the KO mice compared to the WTs. **(C6)** The duration of the longest wake cycle within each 24 h recording were significantly longer in KO mice than in WT mice. ^*^p < 0.05.

Disrupted sleep-wake cycles have been reported clinically in Rett syndrome (Ellaway et al., 2001; Young et al., 2007). Similar assessments in Mecp2-deficient mice have been lacking and significant alterations have recently been reported for Mecp2-deficient heterozygous female mice (Wither et al., 2012). In this study, 24 h EEG/EMG recordings analyzed with confirmation from synchronous video recordings revealed significant sleep dysfunction in Mecp2 KO mice compared to their age-matched WT littermates (Figure 1A). Overall wake and sleep times between WT and KO over 24 h did not show significant differences in wake time percentages for KO compared to WT (62.2 ± 4.6 vs. 55.2 ± 4.4%, respectively) and sleep time percentage (37.4 ± 4.6 vs. 44.5 ± 4.6%, respectively; Figure 1B1). The importance of 24 h recording became evident when activity time were spit by light and dark phase cycles (i.e., bar over Figure 1A hypnograms; 12 h each) where the data showed a highly significant increase in wake times for KO mice during the dark cycle (Figures 1B,C) that were absent during the light cycle.

Rodents cycle between activity states throughout the light and dark cycle in what is called the ultradian cycle. Since rodents are nocturnal creatures, they are more active during the dark cycle than during the light cycle as was evident for WT mice in Figure 1B. Evaluating the periodic cycling between sleep and wake cycles in Mecp2 null mice revealed severe disruption of the normal physiological transition between these activity states. Overall transitions between wake and sleep cycles were significantly lower in KO mice compared to WT (Figures 1C1,C2) when estimated by total number of wake and sleep cycles for the 24 h recording. This was a direct result of significantly longer durations of wake cycles (Figures 1C3,C4). This finding was not significant for sleep cycles. Since all mice in the study were introduced into a novel tethered chamber for the recording period, and heightened anxiety behavior has been documented in Mecp2 null mice (Lang et al., 2014); the first wake cycle durations (i.e., latency to first sleep cycle) were analyzed and found to be longer in KO mice compared to WT (Figure 1C5), however not significantly (*p* = 0.06). In contrast, the longest wake cycles during the 24 h period were typically seen during the dark cycle in KO mice and were significantly longer than wake cycles in WT mice (Figure 1C6, see representative KO hypnograms in Figure 1A).

**Figure 2:**
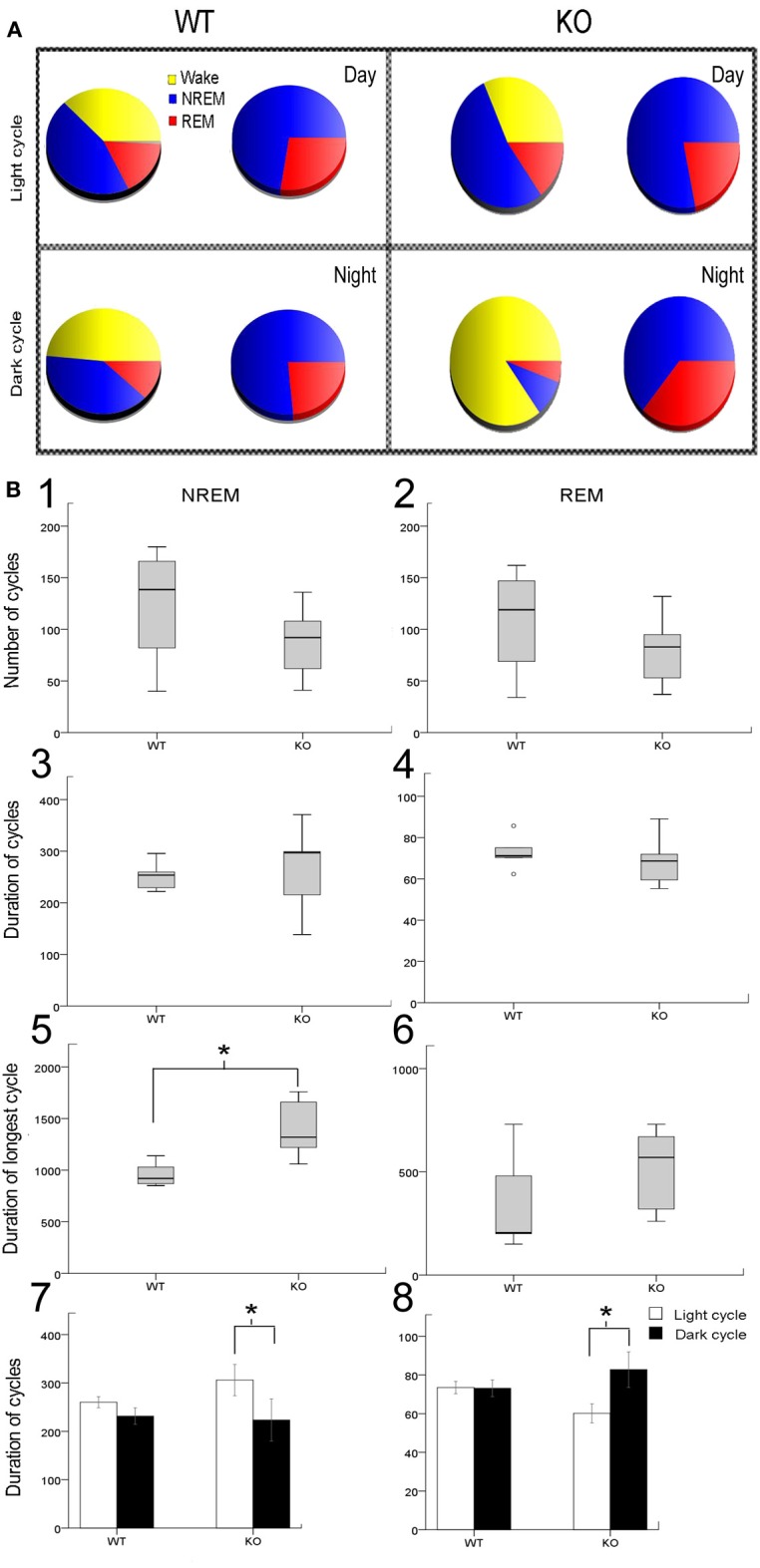
**Sleep microstructure—(A) Representative sleep-wake-cycle pie charts represent the diurnal variation in wake/sleep cycle (yellow, red, and blue) changes in WT vs. KO**. The pie chart on right (red and blue only) highlights the sleep pie charts related to NREM vs. REM within the 12 h activity charts on left. **(B)** Analysis of sleep microstructure cycles showed fewer NREM and REM cycles as expected due to fewer ultradian sleep cycles in KO mice; however this deficit was not significant **(B1,B2)**. In spite of the lower number of NREM and REM events in KO mice, their duration was not significantly different from WT mice **(B3,B4)**. To investigate how KO mice compensated for sleep deprivation, we determined the longest period of NREM and REM sleep. **(B5,B6)** KO mice showed a significantly longer duration of the longest NREM than WT mice. However, the duration of the longest REM cycle was not significantly different in KO and WT mice. **(B7,B8)** Bar graphs for mean group data from representative pie charts in **(A)**. KO mice had a significantly shorter duration of REM sleep cycles during the light cycle compared to WT mice. Additionally, the average duration of REM sleep cycles was significantly longer during the dark cycle than during the light cycle in KO mice that was not detected in WT mice. Average duration of NREMs was not significantly different between WT and KO however a trend toward diurnal variation was detected in KO that was not present in WT mice. ^*^p < 0.05.

The sleep cycle microstructure in the KO mice also showed deviations in the number and duration of cycles that was light cycle-specific (Figure 2A). Severe sleep dysfunction has been reported in Rett patients (Young et al., 2007). A strong relationship between sleep states, particularly REM sleep and learning, has previously been reported (Smith, 1996). Sleep microstructure, as related to NREM and REM cycles, revealed significant impairments particularly related to the light cycles in KO mice that were not seen in the WT mice. The 24 h data analysis showed that the number of NREM and REM cycles (Figures 2B1,B2) were not significantly different from controls. The average durations of NREM and REM cycles were also not significantly different from WT when evaluated over the 24 h recording period (Figures 2B3,B4). However, these differences became significant when looking at longest durations for NREM between KO mice and WTs (Figure 2B5). Longest duration REM cycles did not show significant differences between the WT and KOs (Figure 2B6). More importantly, when the data was analyzed by light vs. dark cycles, sleep microstructure impairments related to both the NREM and REM cycle durations became evident. Significant differences between the light and dark cycle NREM (*p* = 0.009) and REM (*p* = 0.001) cycle durations was detected in KO mice but not seen in the WTs (Figures 2B7,B8; analyses run using a linear mixed model with random mouse effect). This shows that, there was a significant diurnal variation in light and dark cycle NREM and REM durations in the KO mice not detected in the WT littermate mice, indicating that MeCP2 plays an important role in maintaining the sleep microstructure.

### Sleep structure and EEG spectral analysis

**Figure 3:**
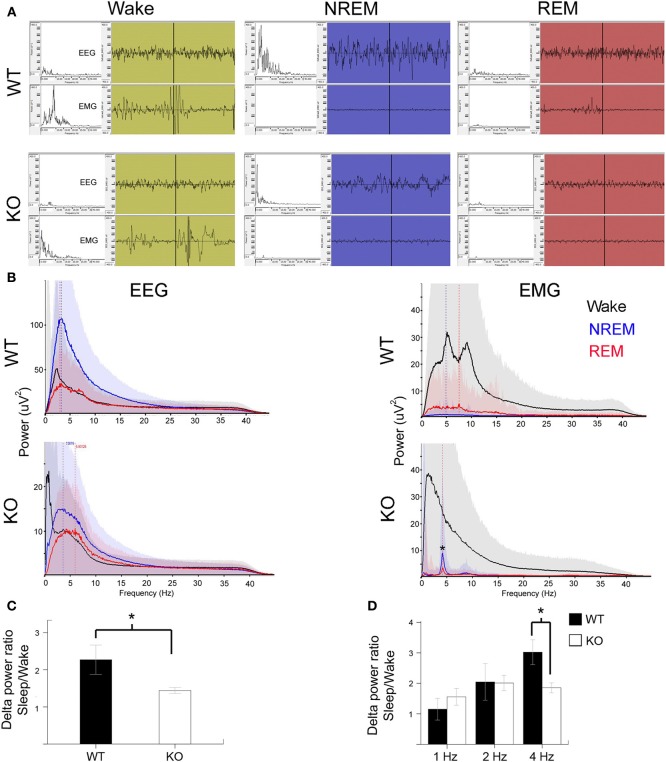
**Poor SWA sleep during NREM indicates severe sleep dysfunction in KO mice. (A)** Representative 10 s EEG traces (top panel) were scored as wake (green), NREM (blue), and REM (red) along with the synchronous EMG (bottom panel) recorded from WT and KO mice. Insets on left are spectrograms for the EEG and EMG for those 10 s in the 0.5–40 Hz range (left to right). EEG spectrogram showed that KO mice had poor power in the delta range (0.5–4 Hz) that is typical of SWA sleep during NREM compared to WT mice. Note WT REM shows a typical muscle twitch artifact on EMG. **(B)** Power analyses for the same mice represented in EEG traces for the 24 h scoring showed a severe overall deficit in NREM power in delta range in KO mice. EMG power showed accurate patterns of associations for the EEG scoring in that the most power was observed during wake states followed by REM when isolated brief muscle twitches were recorded. Note the artifact peak (^*^) in KO EMG during REM and NREM sleep was due to the leads picking up EKG signals when the mouse was sleeping in a hunched position. **(C)** Group data for quantification of delta power during NREM for each mouse normalized over delta power during their individual wake cycles showed significant difference between WT and KO mice in SWA strength. **(D)** This significance was driven by delta activity in the 4 Hz range.

Slow wave-activity (SWA) sleep is characterized by high amplitude low frequency delta wave (0.5–4 Hz) predominance especially during NREM sleep (Figure 3A). REM sleep also known as pardoxical sleep, lasted a few 10's of seconds and resembled wake state EEG in spectral power distribution. However, unlike wake states, EMG was silent during these episodes. Occasionally 1–2 s short muscle twitches were recorded during REM (see representative EMG trace during REM in a WT mouse). Significant attenuation of delta wave power was noted during NREM sleep in KO mice compared to the WT (Figures 3A–C). This finding indicates that in addition to the alterations in the sleep microstructure described in Figure 3, the quality of SWA sleep, which is the hallmark of NREMs, was poor compared to WT littermates. Therefore, not only was there significant dark cycle sleep deprivation in Mecp2 KO mice, the quality of each sleep cycle was also severely impaired (Figures 3B,C). This impairment was driven specifically by significantly low power in the 4 Hz frequency SWA range (Figure 3D) in the KO compared to WT control. This shows that qEEG identified severe and significant deprivation in SWA as one of the biomarkers underlying pathogenesis in symptomatic KO mice.

### Sleep dysfunction and long wake cycles were not associated with a behavioral phenotype

In general, Mecp2 KO mice are universally reported to be hypoactive (Lang et al., 2014). However, none of these reports have looked at the circadian differences with 24 h recordings since rodents are more active nocturnally. To ascertain whether the sleep deprivation ultradian cycles occurring during the dark cycle with long wake cycles were associated with any behavioral dysfunction, we behaviorally scored every 5 min epoch by an activity scale graded to score increasing activity behaviors on video (see methods). One hour time slots of video during wake EEGs, both during the light and dark cycles, were scored every 5 min for the highest activity scores noted based on the scale described in methods. Activity scores (see methods) assigned to video behaviors during wake cycles did not show significant differences between WT and KO mice. Additionally, the data showed that the significantly long wake cycles during the dark phase in KO mice were not associated with any significant hypo- or hyperactivity.

### Activity-dependent glutamate levels disrupted in Mecp2 KO mice

**Figure 4:**
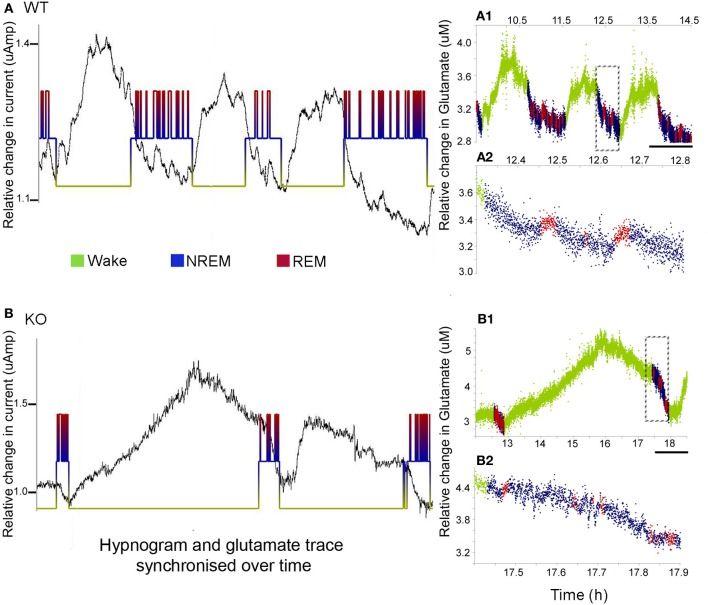
**Sleep cycle dysfunction and glutamate loads. (A,B)** show the synchronous and relative glutamate change associated with the EEG/EMG and video scored hypnograms for representative WT and KO mice respectively [raw glutamate *in-vivo* recording (black) overriding hypnograms (green = wake, blue = NREM, and red = REM) for each mouse]. Both groups showed an activity dependent increase in glutamate during the wake cycle and a decrease during the sleep scored EEG. **(A1,B1)** Color-coding of the rate of glutamate revealed significant differences in the dynamics of the rates of change between KO and WT mice. Scale bar = 1 h. **(A2**,**B2)** Expanded times scales of sleep microstructure [of time slots marked by dotted rectangles in **(A1,B1)**] show the slow decline in glutamate levels as sleep progresses and also the small peaks of glutamate that are tightly correlated with REM cycles (paradoxical sleep) not clear in the KO.

Cortical development involves synaptic formation and elimination. While synaptogenesis predominates earlier and pruning later, the two processes are thought to happen concurrently (Maret et al., 2011). The synaptic homeostasis hypothesis of sleep proposes that slow wave sleep causes downscaling of synaptic networks potentiated during information uptake in prior wakefulness (Born and Feld, 2012). In this study, we found glutamate peaks associated with wake states, and troughs associated with sleep, in both the WT and KO mice (Figure 4). Glutamate concentrations consistently increased with the onset of wake cycles and dropped with the onset of sleep. However, the dynamics of the rate of rise and fall of glutamate were distinctly different between the two genotypes (Figure 4A vs. Figure 4B). Unlike glutamate dynamics detected in WT mice, glutamate levels were observed to start to drop during the extended wake cycles in the KO mice (Figure 4A1 vs. Figure 4B1). The sleep cycle and glutamate dynamics were even tighter with miniature short duration glutamate peaks recorded for every REM cycle (i.e., paradoxical sleep) within each sleep cycle (Figure 4B1 vs. Figure 4B2).

**Figure 5:**
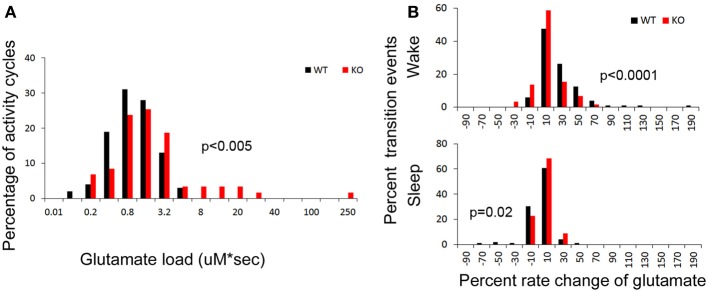
**(A)** Group data for glutamate loads calculated per wake cycle in drift corrected traces of glutamate *in-vivo* recordings (see Methods). Frequency histogram represents glutamate loads per wake (activity) cycle for every mouse in study and show significantly higher number of activity cycles with larger glutamate loads in KO mice compared to WT (red distribution curve shifts to the right, *p* < 0.03). (**B)** Frequency histograms of the percent rates of change of glutamate over time for every transition to wake and sleep states. Both these transitional dynamics were significantly altered in KO mice. The KO mice showed a slower rate of rise (red shift to left) and faster rate of fall (red shift to right) of glutamate that was associated with transitioning to wake and sleep respectively, compared to WT mice (black).

To calculate glutamate loads associated with each wake cycle (i.e., area under curve for each wake cycle), every *in vivo* raw glutamate trace in the study underwent adjustments for the exponential drop during the *in-vivo* calibration phase during the initial 2–3 h of recordings and the linear drifts in some traces during the 24 h recording. Automated analysis calculated the area under each crest associated with each wake cycle. The data demonstrate that in spite of earlier onset glutamate crest falls associated with the longer wake cycles in KO mice, the overall mean glutamate loads associated with wake cycles in KO mice were significantly larger than those in WT mice [Figure 5A, *p* < 0.005]. Since some wake cycles straddled the switch between light to dark cycle and vice versa, data could not be analyzed for diurnal variation in glutamate loads. However, since activity was consistently associated with relatively higher glutamate, it was expected that longer wake cycles associated with the dark cycle would have larger areas under the curve. There was a highly significant correlation between glutamate loads and wake durations in both WT and KO mice [*r* = 0.65 and 0.75 respectively, and *p* (2 tailed) < 0.0001 for both genotypes]. Incomplete wake cycles at the beginning and end of the recording session were not included in this analysis.

**Figure 6:**
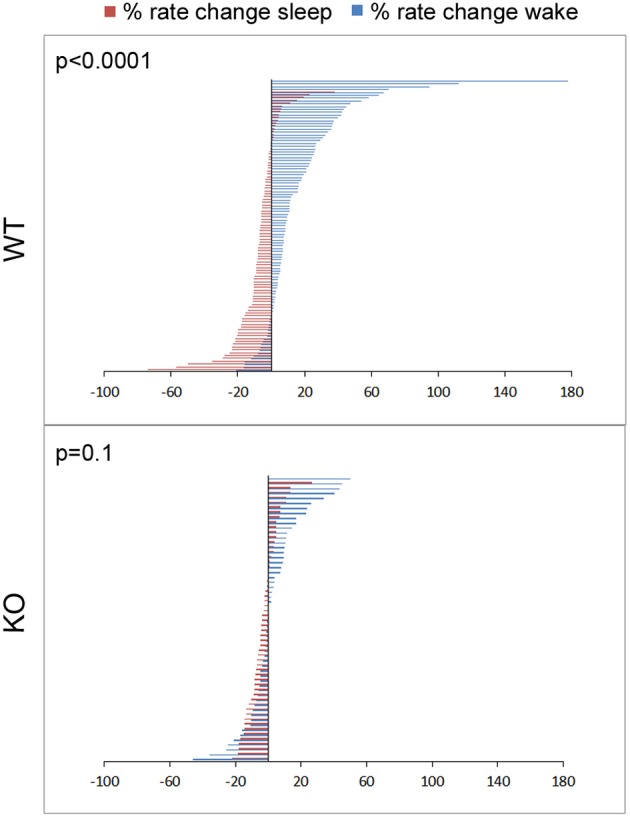
**Physiology of sleep and wake state related glutamate dynamics in WT vs. KO mice**. Percent rate of change of glutamate concentrations in extracellular space following transitions from sleep-wake to wake-sleep states on EEG, happen within minutes. Percent rate of change of glutamate over the first 5 min of transitioning to a sleep or wake state for all sleep-wake cycles in the study (*n* = 103 and 58 WT and KO respectively) were defined by 2 significantly different and opposite glutamate dynamics with very little overlap between the 2 transitions phases in WT mice. This difference was obliterated in KO mice due to dysfunction of the activity dependent glutamate homeostasis that distinguished the dynamics between the 2 transition phases and genotypes at 7 weeks old.

Dynamic and progressive changes in the concentration of glutamate that correlated significantly as a function of behavioral state as scored on EEG/EMG were noted. These changes were independent of time of day but dependent on the sleep cycle state. Therefore, rate of change in glutamate rise and fall as related to onset of each wake and sleep state were calculated to evaluate glutamate homeostasis associated with the transitional zones between the wake and sleep states (Figure 5B). Specifically, the concentration of glutamate increased consistently and progressively on waking (17.03 ± 2.7%/5 min) and REM sleep (1.04 ± 0.48 %/10 s). This increase was counteracted by a progressive decrease in relative glutamate levels during NREM sleep (−8.17 ± 1.42 %/5 min) in WT mice. When activity dependent glutamate homeostasis was evaluated in KO mice, a severe disruption was noted. Mean percent rate of increase of glutamate with wake was 2.5 ± 2.4%/5 min and significantly slower than WT (*p* < 0.0001). Similarly, mean percent rate of fall of glutamate with sleep onset was −3.26 ± 1.24%/5 min and also significantly slower than WT (*p* = 0.02). Comparing glutamate homeostasis within WT mice for transition to wake vs. sleep states showed the dynamics were significantly different (*p* < 0.0001; Figure 6). This difference in transitional states glutamate homeostasis was completely obliterated in the KO mice (*p* = 0.1; Figure 6). The restorative function of sleep has been recently proposed to be a consequence of the enhanced removal of potentially neurotoxic waste products that accumulate in the awake central nervous system (Xie et al., 2013). The significantly slower rate of fall of glutamate with sleep onset in KO mice supports this hypothesis.

### Absolute brain glutamate levels are higher in frontal cortex of KO mice

**Figure 7:**
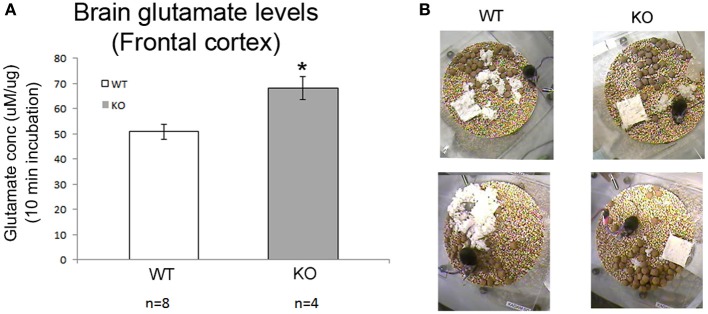
**Absolute brain glutamates levels and sleep deprivation a vicious cycle. (A)** Brain glutamate levels in frontal cortex were significantly higher in KO mice compared to WT. **(B)** Show poor nesting behavior scoring as detected in our 24 h video recordings where nesting pads were left untouched overnight (^*^p < 0.05).

Brain glutamate levels measured in frontal cortex of WT and KO mice at 7 weeks of age (both from recorded and naïve mice) were significantly higher in KO mice than in WT controls (Figure 7A, *p* = 0.008).

### Chronic sleep deprivation and developmental degeneration

Weights of the WT (male and female), KO males and HET females in the breeding colony was recorded over 7 weeks, demonstrated that HET mice showed no weight lag compared to age-matched WT females in the period when they are not symptomatic. However, KO males start to show a weight lag at 3 weeks of age when compared to WT males that was initially not significant but became significant at 7 weeks of age. Twenty-four hour videos associated with the EEG recording also revealed the same poor nesting behaviors in symptomatic KO mice compared to the WT (Figure 7B) that have been previously reported. Mice when placed in individual recording chambers were provided with a nest-building material (5 × 5 × 0.5 cm). Every mouse got a new material pad at the beginning of the 24 h recording, and nesting ability was scored overnight based on the interaction of individual mouse with the nesting material on a graded scale previously described. KO mice scored poorly with nesting since they did not interact with the material at all, WT mice completely used the material to build a well-structured nest (*p* = 0.02), matching previously reported studies in this mouse model.

## Discussion

This study detected novel EEG and *in-vivo* glutamate homeostasis biomarkers that clearly differentiated a symptomatically progressive KO mouse from its age-matched WT littermate. Firstly, we found that symptomatic KO mice had severe sleep dysfunction that was circadian- and activity cycle-dependent, and could be reliably quantitated by the activity state scoring of 24 h continuous EEGs. The resultant hypnograms represented a chronic sleep deprivation-like profile that was not followed by compensation. Secondly, the sleep microstructure and quality of SWA during NREM was also consistently and significantly altered in the symptomatic KO mice. Impaired NREM and REM dynamics and poor SWA were biomarkers in the symptomatic KO mice during the progressive deterioration phase that ends in early mortality in this model. Thirdly, activity-dependent glutamate homeostasis was observed in both WT and KO, but KO mice showed a significant increase in activity cycle-associated glutamate loads associated with the long wake cycles. Since these abnormal activity-dependent glutamate changes were relative to wake/sleep state and did not represent the actual base load, we ascertained absolute glutamate concentrations in the frontal cortices. Glutamate levels measured from fresh frozen brain tissue of recorded and naïve KO mice at 7 weeks of age were significantly higher than age-matched WT littermates. Together these findings suggest that chronically elevated glutamate levels may underlie the disease progression in this mouse model of Rett syndrome. Finally, glutamate homeostatic mechanisms associated with transitions to and from wake and sleep states, were severely disrupted in the symptomatic KO mice. The percent rate of rise and fall of glutamate was tightly tied to activity states as determined by EEG. The dynamics of this change of glutamate concentrations associated with transitions between waking and sleep states were severely and significantly altered in the KO brains *in-vivo*. Specifically, the distinct and significant differences between the dynamics of the rates of rise of glutamate associated with wake and rates of fall associated with sleep onset detected in WT were obliterated in KO mice, which suggested severe homeostatic dysfunction. In summary, we found a triad of sleep disorder biomarkers in KO mice that represented both a disintegrated ultradian rhythm and a dark cycle aggravation that could underlie the higher absolute glutamate concentrations detected in the KO forebrains. Chronic sleep architecture dysfunction and poor sleep quality in KO mice could be one of the mechanisms underlying regression in weight gain, poor nesting and poor learning memory in these mice (Pelka et al., 2006).

Recent evidence suggests that alterations in cortical glutamatergic synaptic responses and excitatory connectivity may play an important role in RTT, but studies vary in whether the balance between cortical excitation and inhibition is shifted in favor of inhibition (Dani et al., 2005) or hyperexcitability (Medrihan et al., 2008; Zhang et al., 2008). Other studies show that the loss of Mecp2 function lead to the higher susceptibility of primary neuronal cultures to excitotoxicity (Russell et al., 2007; Fischer et al., 2009), however these studies were not conducted in the frontal cortex. The now-established prominent role of glia in RTT pathogenesis (Maezawa et al., 2009; Maezawa and Jin, 2010; Lioy et al., 2011) may contribute to excitotoxicity through impaired glutamate re-uptake. The consistent emerging picture with Mecp2 deletion is that there is a regional specificity to the Mecp2 dynamics between glial and neuronal cells that cannot be generalized for the entire brain (Lioy et al., 2011; Kang et al., 2014). Little is known as to how synaptic excitability alters as a function of the clinical stages described in RTT (Glaze and Schultz, 1997). Sleep cycles are known to play crucial roles in these processes as the sleep hypnograms evolve in the maturing brain. These secondary effects, occurring in association with the genetic disruptions of synaptic plasticity in RTT (also recently shown in a model for Down's syndrome; Kaur et al., 2014) may exacerbate the disease progression and play a significant role in intellectual disabilities. Therapies directed at correcting these disruptions may provide a novel mechanism by which to curb disease progression.

In the KO mice, disruption of the ultradian cycle specifically during the dark cycle denoted by long wake periods represented a sleep deprivation like profile. As sleep pressure increased, glutamate concentrations ceased to increase and began to decrease despite continuous waking. In spite of this, glutamate load during the wake periods was significantly larger than in WT mice. In addition, the sleep cycles following the abnormally long wake cycles did not exhibit the natural compensatory deep sleep homeostatic patterns that are known to counteract acute sleep deprivation in WT mice. However, it has been shown that when animals are chronically sleep deprived compensatory mechanisms begin to fail even in WT rodents (Kim et al., 2007).

With Mecp2 KO mice, the long wake cycles specific to the dark phase and the associated higher baseline absolute brain glutamate levels in the frontal cortex may indicate that the chronic sleep deprivation like profile of the hypnograms may additionally feed into the chronic elevation of glutamate levels in the frontal cortices of the symptomatic KO mice. Chronic sleep deprivation can lead to chronically elevated glutamate levels in the extrasynaptic space, which may be one of the reasons of the higher levels detected in the CSF of RTT patients (Lappalainen and Riikonen, 1996; Horska et al., 2009). The resulting raised glutamate itself then can act through extrasynaptic glutamate receptors and disrupt brain function and sleep structure (Hardingham and Bading, 2010). This vicious cycle could be one of the mechanisms that underlie the regressive stages in the lifespan of Mecp2 KO mice. We also found that the glutamate homeostasis associated with the wake and sleep states was severely affected in KO mice. These two findings may underlie a vicious cycle that leads to the early fatality in KO mice. Activity-dependent neuronal firing patterns, neuromodulators, and cerebral metabolism modulate the synaptic release of glutamate and therefore extrasynaptic glutamate. Extrasynaptic glutamate in turn can affect neural function and may be neurotoxic.

Very few studies have investigated how or whether extracellular glutamate is regulated across sleep-waking states (Dash et al., 2009). Dash et al. have shown in rats that the progressive increase in cortical extrasynaptic glutamate during EEG-activated states (wake) is counteracted by a decrease during NREM sleep that is additionally modulated by sleep pressure following sleep deprivation. Our studies provide evidence for a long-term failure of homeostasis of extracellular glutamate across sleep-waking states in KO mice. Disruption of this homeostasis might allow for higher extrasynaptic glutamate levels over longer periods without compensation that may be toxic over a period of time. However, our *in-vivo* investigation was limited to the frontal cortex, and the findings reported here may depict glutamate-related biomarkers specific to that region only. Although in the Mecp2 KO mice (Mecp2^tm1.1Bird/y^) examined in this study, the MeCP2 is missing from the entire brain cell population, there is evidence that it still results in location- and temporal-specific alterations in synaptic physiology (Kang et al., 2014). This may also indicate that the role of Mecp2 in synaptic physiology in health and disease is determined by location and age (Lioy et al., 2011).

The EEG pattern of NREM sleep is dominated by SWA. SWA is defined as the EEG power in the 0.5–4 Hz band during NREM sleep, and is a reliable measure of the amplitude and number of slow waves during this phase of sleep. SWA is an established marker of sleep pressure, increasing with the duration of waking and dissipating throughout sleep. Sleep deprivation studies in WT rodents and humans have shown a tendency toward NREM compensation by increase in SWA during sleep cycles following sleep periods of sleep deprivation. With the inherent sleep-deprivation-like profile detected in all KO hypnograms, not only did the NREMs following the long wake cycles fail to show any compensatory increase in SWA, in contrast, the poor SWA NREMs typical to the KO mice persisted following the long wake cycles. These impairments were statistically significant. Mecp2 KO mice showed a severe impairment in glutamate homeostasis associated with SWA during NREM. Glutamate levels decreased at a faster rate during NREM sleep periods with high SWA than during those with low SWA. In rats the rate of decrease in glutamate during sleep after sleep deprivation was twice as fast as the average decrease during spontaneous NREM sleep (Dash et al., 2009). High sleep pressure seems to enhance the NREM-related decline in glutamate levels in naïve brains that are sleep deprived. However, we found that the rate of fall of glutamate during NREM following long wake cycles in Mecp2 KO mice was actually slower than the rate of fall in their age-matched non-sleep deprived WT littermates. Additionally, although no frank generalized EEG seizure activity was detected with the 24 h recording protocol in any of the KO mice, epileptiform spike wave discharges (SWD) were detected in KO mice, similar to previous reports (Ward et al., 2011; Lang et al., 2014).

## Conclusion

Chronic impairment of sleep and glutamate homeostasis underlies the regression in symptomatic Mecp2 KO mice. Continuous 24 h EEG and glutamate recordings allow quantification of the impairment, which can be used as biomarkers of regression. Recent work using viral delivery of Mecp2 cDNA has shown promise by significantly ameliorating the disease in female mutant mice (Garg et al., 2013) and in male nulls (Gadalla et al., 2013) based solely on behavioral outcomes. However, the effect of ectopic transduction of MeCP2 as therapy for the sleep and glutamate homeostatic markers of disease progression remains unknown, and future studies could integrate this technology to investigate the functional rescue of CNS networks. Additionally, novel intervention therapies can now be tested to evaluate efficacy in rescuing these *in-vivo* biomarkers to ascertain effect on modulating regression and preventing fatality.

### Conflict of interest statement

The authors declare that the research was conducted in the absence of any commercial or financial relationships that could be construed as a potential conflict of interest.

## Supplementary material

Figure S1 **Experimental design and biosensor glutamate specificity. (A)** Schematics of biosensor that used glutamate specific oxidative enzyme reactions to detect every molecule of glutamate in extracellular space where implanted (image with permission from vendor website). **(B)** Schematic of location of stereotaxic implant to biosensor into frontal neocortex (blue); placement of EEG leads (2 recording and 1 reference) and mounting screw to anchor head mount to skull. **(C)** Representative post-recording calibration trace of recording shows specificity to glutamate. Step readings for every 10 uM glutamate (three repeats) added to media were averaged for each sensor in this study (time scale = 1 min). Inset graph shows tight correlation of vendor generated pre-calibration values for glutamate before implant compared our 24 h post-recording calibrations. This allowed us to estimate post-calibration values for biosensors damaged during extraction from brain (red dots). **(D)** *In-vivo* glutamate biosensor recording shows response to an IP injection of MK801 to a WT mouse in study. Rapid increases in glutamate levels in trace after injection artifact (arrowhead) indicate potent NMDA receptor block and sensitivity of biosensor *in-vivo* (time scale bar = 5 min).

Figure S2 **Representative manual EEG scoring for behavioral state resulting in a 24 h hypnogram and the associated automated EEG and EMG power. (A)** shows three traces of EMG power (top black), genotype blinded and manually scored EEG scoring generated hypnogram (middle) and automated EEG power (bottom blue) for a single 24 h recording. **(A1)** Shows an expanded time scale for duration of recording in **(A)** marked by top blue bar. Black arrows show transition states between wake into sleep and vice-versa that are closely tied to the EMG power trace that is activity dependent. EEG power in blue shows the opposite trend to the EMG power trace, with high power during NREM sleep due to the dominance of high amplitude slow wave-activity (0.5–4 Hz SWA) forms in NREM. The dotted lines highlight the drop in EEG power associated with onset of every REM cycle within a sleep cycle (paradoxical sleep). The automated EEG and EMG power alignment to the manually conducted EEG scoring in this study validates the efficacy of the behavioral state EEG used to quantitate the sleep architecture.

## Abbreviations

EEG: electroencephalogram
Mecp2: methyl CpG binding protein 2
RTT: Rett syndrome
EMG: electromyogram
REM: rapid eye movement sleep
NREM: non-rapid eye movement sleep
WT: wild type
KO: knock out.
