# Supplementary figures and images for: Twenty-four hour quantitative-EEG and in-vivo glutamate biosensor detects activity and circadian rhythm dependent biomarkers of pathogenesis in Mecp2 null mice
Source: Front Syst Neurosci. 2014 Jun 27;8:118. doi: 10.3389/fnsys.2014.00118 (PMC4072927; doi:10.3389/fnsys.2014.00118)

A

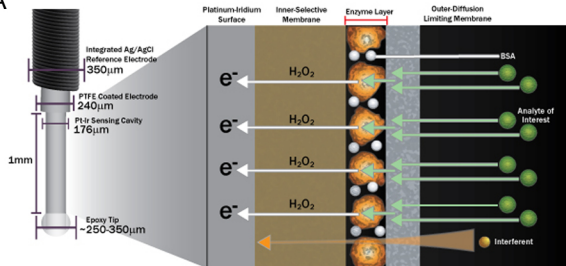

B

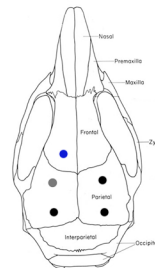

Biosensor  
EEG electrodes  
Ground

C

Glutamate change (uAmps)

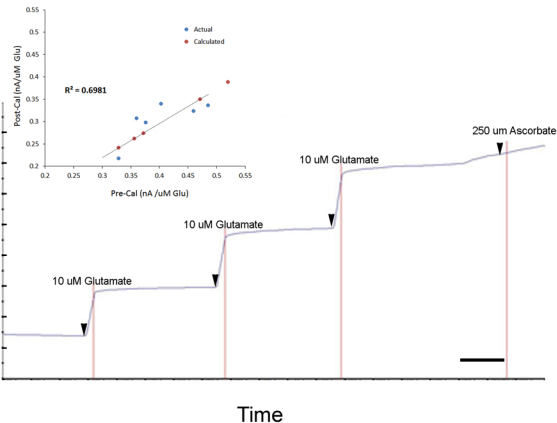

D

Glutamate change (uAmps)

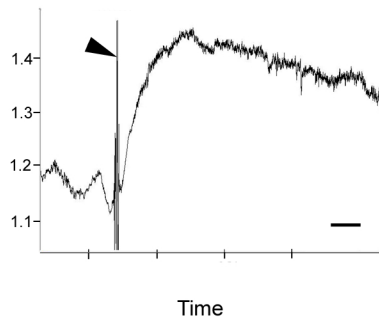

Supplement: Figure S1 — Experimental design and biosensor glutamate specificity. (A) Schematics of biosensor that used glutamate specific oxidative enzyme reactions to detect every molecule of glutamate in extracellular space where implanted (image with permission from vendor website). (B) Schematic of location of stereotaxic implant to biosensor into frontal neocortex (blue); placement of EEG leads (2 recording and 1 reference) and mounting screw to anchor head mount to skull. (C) Representative post-recording calibration trace of recording shows specificity to glutamate. Step readings for every 10 uM glutamate (three repeats) added to media were averaged for each sensor in this study (time scale = 1 min). Inset graph shows tight correlation of vendor generated pre-calibration values for glutamate before implant compared our 24 h post-recording calibrations. This allowed us to estimate post-calibration values for biosensors damaged during extraction from brain (red dots). (D) In-vivo glutamate biosensor recording shows response to an IP injection of MK801 to a WT mouse in study. Rapid increases in glutamate levels in trace after injection artifact (arrowhead) indicate potent NMDA receptor block and sensitivity of biosensor in-vivo (time scale bar = 5 min). [file Presentation1.PDF]

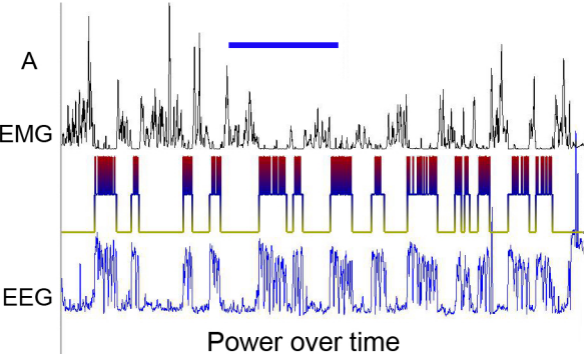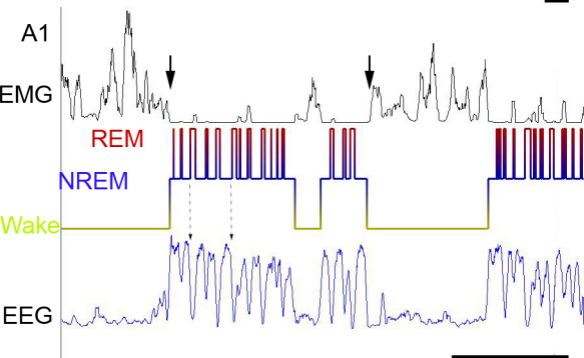

Supplement: Figure S2 — Representative manual EEG scoring for behavioral state resulting in a 24 h hypnogram and the associated automated EEG and EMG power. (A) shows three traces of EMG power (top black), genotype blinded and manually scored EEG scoring generated hypnogram (middle) and automated EEG power (bottom blue) for a single 24 h recording. (A1) Shows an expanded time scale for duration of recording in (A) marked by top blue bar. Black arrows show transition states between wake into sleep and vice-versa that are closely tied to the EMG power trace that is activity dependent. EEG power in blue shows the opposite trend to the EMG power trace, with high power during NREM sleep due to the dominance of high amplitude slow wave-activity (0.5–4 Hz SWA) forms in NREM. The dotted lines highlight the drop in EEG power associated with onset of every REM cycle within a sleep cycle (paradoxical sleep). The automated EEG and EMG power alignment to the manually conducted EEG scoring in this study validates the efficacy of the behavioral state EEG used to quantitate the sleep architecture. [file Presentation2.PDF]
